# Plant Cysteine Oxidase Oxygen-Sensing Function Is Conserved in Early Land Plants and Algae

**DOI:** 10.1021/acsbiomedchemau.2c00032

**Published:** 2022-08-15

**Authors:** Leah J. Taylor-Kearney, Samuel Madden, Jack Wilson, William K. Myers, Dona M. Gunawardana, Elisabete Pires, Philip Holdship, Anthony Tumber, Rosalind E. M. Rickaby, Emily Flashman

**Affiliations:** † Chemistry Research Laboratory, University of Oxford, 12 Mansfield Road, Oxford, OX1 3TA, United Kingdom; ‡ Inorganic Chemistry Laboratory, University of Oxford, South Parks Road, Oxford OX1 3QR, United Kingdom; § Department of Earth Sciences, University of Oxford, South Parks Road, Oxford OX1 3AN, United Kingdom

**Keywords:** enzyme kinetics, evolution, hypoxia, N-degron pathway, oxidase, post-translational modification

## Abstract

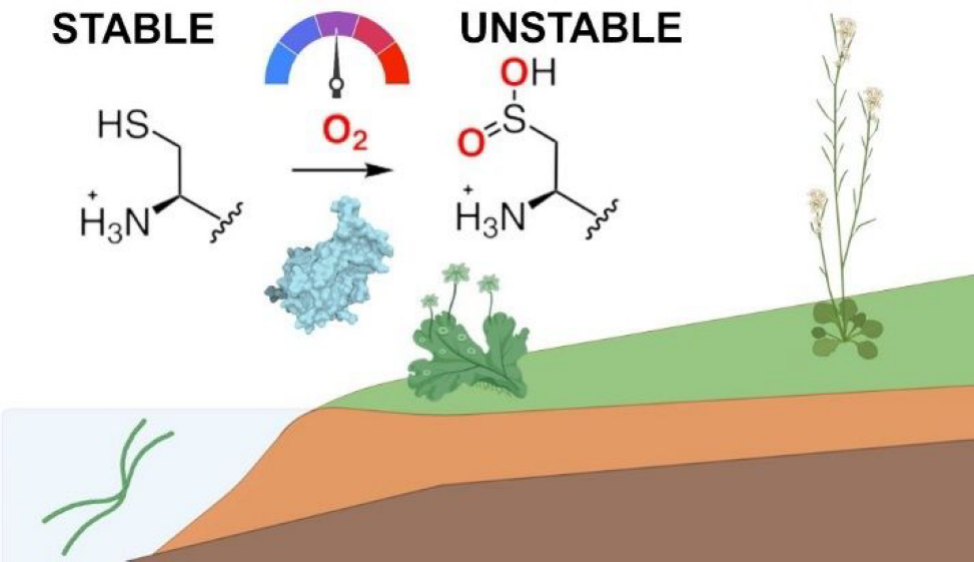

All aerobic organisms require O_2_ for survival. When their O_2_ is limited (hypoxia), a response is required to reduce demand and/or improve supply. A hypoxic response mechanism has been identified in flowering plants: the stability of certain proteins with N-terminal cysteine residues is regulated in an O_2_-dependent manner by the Cys/Arg branch of the N-degron pathway. These include the Group VII ethylene response factors (ERF-VIIs), which can initiate adaptive responses to hypoxia. Oxidation of their N-terminal cysteine residues is catalyzed by plant cysteine oxidases (PCOs), destabilizing these proteins in normoxia; PCO inactivity in hypoxia results in their stabilization. Biochemically, the PCOs are sensitive to O_2_ availability and can therefore act as plant O_2_ sensors. It is not known whether oxygen-sensing mechanisms exist in other phyla from the plant kingdom. Known PCO targets are only conserved in flowering plants, however PCO-like sequences appear to be conserved in all plant species. We sought to determine whether PCO-like enzymes from the liverwort, *Marchantia polymorpha* (MpPCO), and the freshwater algae, *Klebsormidium nitens* (KnPCO), have a similar function as PCO enzymes from *Arabidopsis thaliana*. We report that MpPCO and KnPCO show O_2_-sensitive N-terminal cysteine dioxygenase activity toward known AtPCO ERF-VII substrates as well as a putative endogenous substrate, MpERF-like, which was identified by homology to the *Arabidopsis* ERF-VIIs transcription factors. This work confirms functional and O_2_-dependent PCOs from Bryophyta and Charophyta, indicating the potential for PCO-mediated O_2_-sensing pathways in these organisms and suggesting PCO O_2_-sensing function could be important throughout the plant kingdom.

## Introduction

**Scheme 1:**
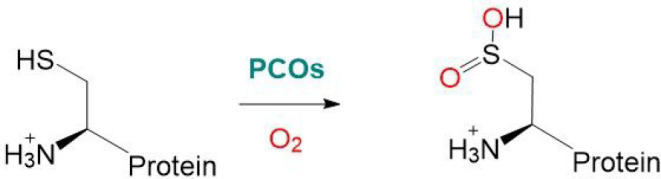
Plant Cysteine Oxidase (PCO)-Catalyzed Oxidation of Protein N-Terminal Cysteine to Cys-Sulfinic Acid

O_2_ is a molecule that has shaped evolution.^1,2^ Across modern surface environments, a range of niches of varying degrees of oxygenation persist. Such evolutionary, temporal, and spatial variability in oxygenation likely requires both long- and short-term organismal adaptation to O_2_ availability. The primary mechanism by which higher plants sense and adapt to low O_2_ availability has been established over recent years.^3−5^ O_2_-sensing enzymes, the plant cysteine oxidases (PCOs), catalyze oxidation of cysteine to Cys-sulfinic acid at the N-termini of target proteins (Scheme 1), a reaction for which the rate is dependent on the availability of molecular O_2_.^6−8^ Co-translational methionine cleavage exposes the N-terminal Cys for oxidation, leading to the degradation of the target protein via the Cys/Arg branch of the N-degron pathway:^9,10^ Oxidized N-terminal Cys residues are substrates for arginyl transferase enzymes, with the arising arginylated N-termini recognized by ubiquitin ligases. Ubiquitination signals for the protein to be degraded by the proteasome.^11,12^ This pathway therefore connects O_2_ availability and destabilization of target proteins, while in low O_2_ (hypoxic) conditions these proteins remain stable due to reduced PCO activity.

PCO target proteins, identified in *Arabidopsis*, include the Group VII ethylene response factors (ERF-VIIs) which are transcription factors involved in responses to submergence-induced acute hypoxia and are defined by their MCGGAI N-termini.^9,10^ Also identified to date are proteins related to plant development: Vernalisation 2 (AtVRN2), involved in cold-induced suppression of flowering,^13^ and Little Zipper 2 (AtZPR2), which regulates activity of the hypoxic shoot meristem.^14^ Each of these O_2_-signaling mechanisms regulated by the PCOs is arguably unique to flowering and perhaps seeding plants, with evolutionary analysis of substrates limiting their presence to angiosperms and some spermatophytes.^15^ However, PCO-like sequences are conserved in early land plants and algae,^15^ while a homologous enzyme, HsADO, has been reported as regulating the stability of N-terminal Cys-initiating proteins in humans.^16^ This suggests that Nt-Cys dioxygenase function may be an evolutionary conserved mechanism of O_2_ sensing, albeit with different target proteins.^17^

To investigate this possibility, we have expressed PCO-like enzymes from *Marchantia polymorpha*, representing early land plants, and the filamentous freshwater algae *Klebsormidium nitens* (also known as *Klesormidium flaccidum*). *M. polymorpha* is a liverwort, whose ancestor is reputed to be the first land plant and which possibly retains features of both its algal ancestors and extant land plants.^16^*K. nitens* is an undifferentiated semiterrestrial freshwater alga and is a model for understanding the early transition to land.^17^ We show that the MpPCO and KnPCO enzymes are functionally homologous to the *Arabidopsis* PCOs and identify a putative endogenous substrate for MpPCO. We show that MpPCO and KnPCO enzyme activity is O_2_-dependent, with KnPCO activity being highly sensitive to changes in O_2_ availability. Crucially, both enzymes demonstrate the potential, at least biochemically, for a conserved O_2_-sensing function in early plants, raising the possibility that O_2_ sensing is important in the life of aquatic and early land plants.

## Results

### Identification, Purification, and Characterization of MpPCO and KnPCO

MpPCO and KnPCO sequences were identified through BLASTp searches of available proteome and genome portals using AtPCO1–5 as input sequences. Both species contained only one PCO-like sequence, rather than multiple sequences observed in higher plants.^15^ Sequence alignment of MpPCO and KnPCO revealed that they are highly conserved with those of the AtPCOs. MpPCO shares the greatest homology with AtPCO4 at 49.6% identity, while KnPCO shares 43.5% identity with AtPCO1. KnPCO and MpPCO retain key residues relevant to structure and function, for example three iron-binding His residues and conserved Asp and Tyr residues close to the active site known to be important for PCO catalytic activity^18^ (Supplementary Figure S1).

Expression and purification of MpPCO and KnPCO followed protocols previously described for the AtPCOs.^7^ Recombinant proteins were purified via Ni-affinity chromatography and size exclusion chromatography to >95% purity as judged by SDS-PAGE (Supplementary Figure S2A). AtPCOs copurify with substoichiometric levels of Fe (∼0.3 Fe atoms/molecule),^8^ and inductively coupled plasma mass spectrometry (ICP-MS) analysis of MpPCO and KnPCO samples revealed that these enzymes also bind Fe at substoichiometric levels, at 0.16 (±0.006) and 0.27 (±0.013) Fe atoms/molecule, respectively (Supplementary Table S1). These values were used to determine the proportion of active enzyme for subsequent assays. Fe(III) content was similar to the small proportion reported to be present in AtPCO4^8^ (Supplementary Figure S2B); therefore, most of the Fe was assumed to be in the Fe(II) form. Similar to AtPCO4,^8^ both MpPCO and KnPCO also bound Ni (a contaminant of Ni-affinity chromatography) and a low proportion of Zn (Supplementary Table S1).

### MpPCO and KnPCO are Cysteinyl Dioxygenases

**Figure 1:**
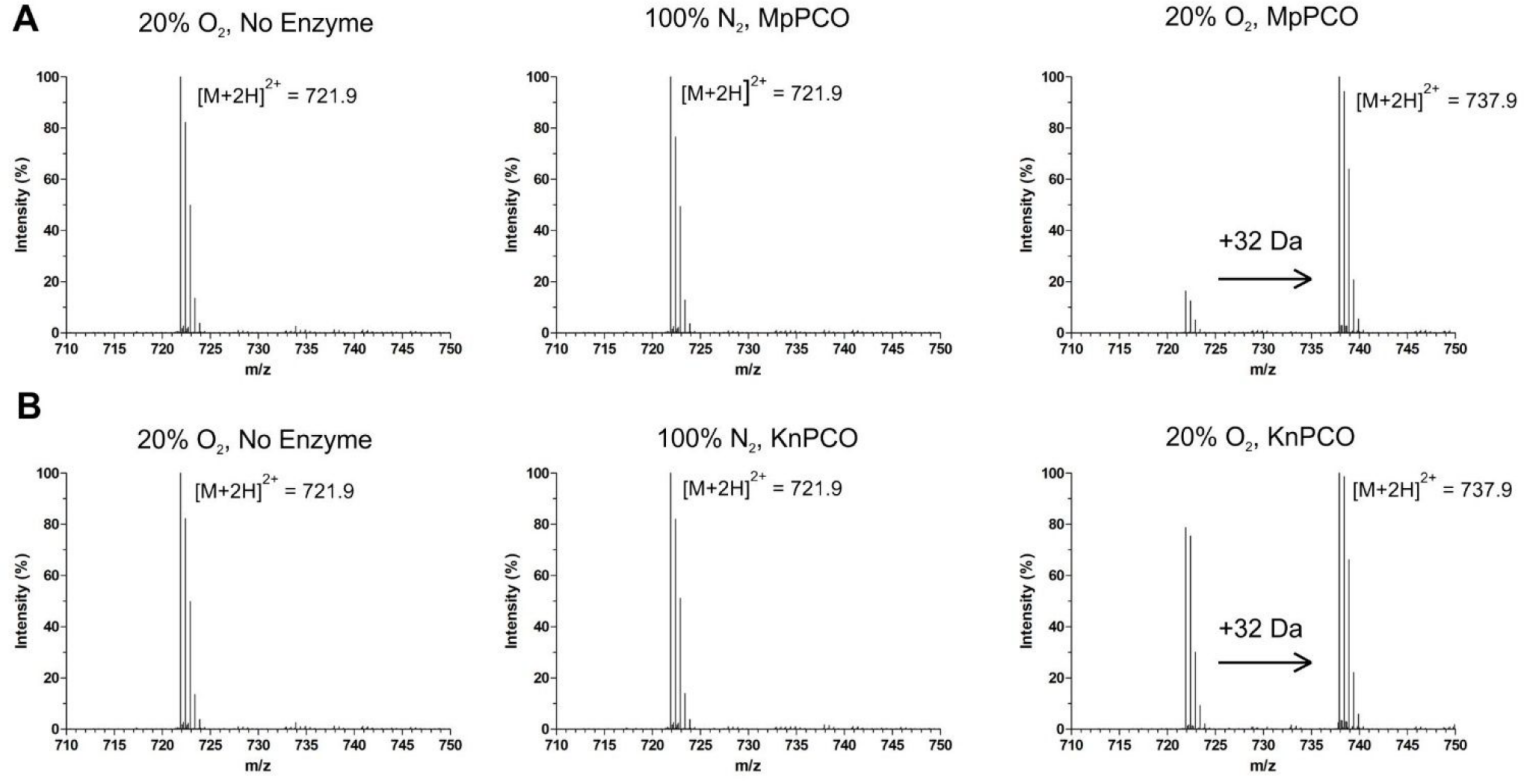
MpPCO and KnPCO catalyze O_**2**_-dependent oxidation of AtRAP2_2–15_. Mass spectra of AtRAP2_2–15_ peptide incubated with and without (A) MpPCO or (B) KnPCO under 20% O_2_ and under 100% N_2_ show a +32 Da increase in the presence of enzyme and O_2_, consistent with N-terminal Cys oxidation. Assays were conducted for 1 h at 25 °C in the presence of 200 μM AtRAP2_2–15_ using 50 mM Tris/HCl, 0.4 M NaCl, 1 mM TCEP, pH 7.5 as buffer.

To determine whether MpPCO and KnPCO have equivalent functionality to the AtPCOs, both enzymes were incubated with a known AtPCO substrate, a 14-mer peptide representing the N-terminus of the *A. thaliana* ERF-VIIs RAP2.2 and RAP2.12 (herein referred to as AtRAP2_2–15_). Following 1 h incubation under atmospheric conditions at 25 °C and subsequent analysis of the mass of the AtRAP2_2–15_ substrate by UPLC-MS, a +32 Da shift was observed in the presence of both MpPCO and KnPCO (Figure 1). This was also dependent on the presence of O_2_, as modification did not take place in the presence of 100% N_2_ under otherwise equivalent conditions. Tandem MS/MS indicated that the modification observed in the presence of O_2_ was localized to the N-terminal Cys residue (Supplementary Figure S3). These data are consistent with MpPCO- and KnPCO-catalyzed oxidation of AtRAP2_2–15_ Nt-Cys to Cys-sulfinic acid, as observed for all of the AtPCOs.^8^ This demonstrates that both MpPCO and KnPCO are cysteinyl dioxygenases, verifying that, at least in vitro, functional PCO enzymes are conserved in early land plants and algae.

### MpPCO and KnPCO Catalyze Oxidation of Known AtPCO Substrates from Angiosperms

**Figure 2:**
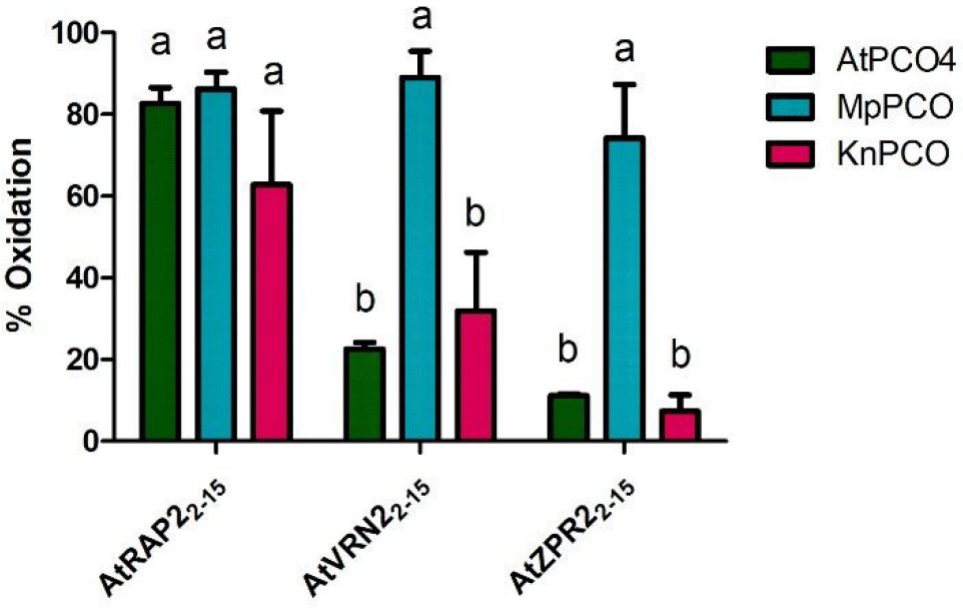
AtPCO4, MpPCO, and KnPCO activity with *A. thaliana* PCO substrates: A 1 h end point assay with peptides representing *A. thaliana* PCO substrates AtRAP2_2–15_, AtVRN2_2–15_ and AtZPR2_2–15_. Assays were conducted at 25 °C using 50 μM peptide, 50 mM bis-tris propane, 50 mM NaCl, and 1 mM TCEP, pH 8.0, as buffer. Letters indicate statistically significant differences (two-way ANOVA, *p* < 0.0001, *n* = 3).

The AtPCOs are known to regulate the stability of two other Nt-Cys initiating substrates, AtZPR2 and AtVRN2.^13,14^ Although, like the ERF-VIIs, these substrates are restricted to flowering plants, we nevertheless sought to determine whether peptides representing the N-termini of these substrates could act as substrates for MpPCO and KnPCO. Peptides representing the Cys-initiating N-termini of each of these substrates (herein referred to as AtVRN2_2–15_ and AtZPR2_2–15_) were incubated for 1 h in the presence of MpPCO, KnPCO and AtPCO4. For each enzyme, oxidation of all three substrates was observed (Figure 2), albeit to differing degrees. AtPCO4 showed considerably greater activity toward AtRAP2_2–15_ (82.6% oxidation) than to AtVRN2_2–15_ (22.6% oxidation) or AtZPR2_2–15_ (11.1% oxidation). MpPCO was most active toward all substrates, showing 86.2% oxidation toward AtRAP2_2–15_, 89.1% oxidation toward AtVRN2_2–15_, and 74.2% oxidation toward AtZPR2_2–15_. KnPCO showed a substrate selectivity profile similar to that of AtPCO4, with greater activity toward AtRAP2_2–15_ (62.8% oxidation) than to AtVRN2_2–15_ (31.9% oxidation) or AtZPR2_2–15_ (7.3% oxidation).

These data indicate that PCO enzymes from different organisms have different substrate selectivity profiles under the conditions tested, with MpPCO showing a particularly robust activity profile toward each substrate. It is important to consider that these are prolonged assays, the results of which may simply reflect the ability of each enzyme to sustain catalytic activity toward each substrate; notably these assays were not supplemented with additional Fe(II) or ascorbate (see below). Furthermore, the proteins represented by the RAP2, ZPR2, and VRN2 peptides are only found within the flowering plants^15^ and thus cannot represent endogenous substrates for MpPCO or KnPCO. Nevertheless, these results do suggest that PCO enzymes from different organisms could demonstrate divergent substrate selectivity.

### MpPCO and KnPCO Catalyze Oxidation of a Peptide Representing an Nt-Cys Initiating Protein from *M. polymorpha*, “MpERF-like”

Given that the substrates tested so far are not physiologically relevant in *M. polymorpha* or *K. nitens*, we next used the online tool Phytozome^19^ to probe proteomic data from these organisms to ascertain whether there are any Nt-Cys-initiating sequences with potential homology to known AtPCO substrates. One Met-Cys initiating protein similar to the AtERF-VII substrates was identified in the *M. polymorpha* proteome (transcript ID: Mapoly0293s0001). The protein is a putative AP2/ERF-like transcription factor and, when compared with the AtERF-VII substrates, shares the highest percentage identity with the AtERF-VII Hypoxia Response Element 2 (AtHRE2) at 32.8%. We refer to this protein as “MpERF-like” to reflect this homology.

**Figure 3:**
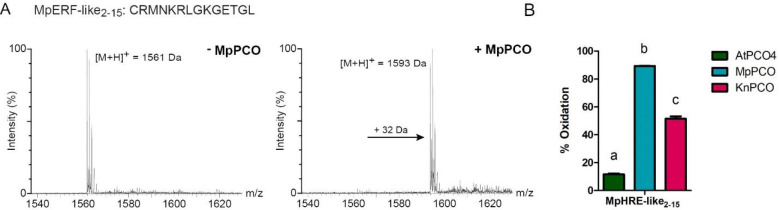
MpPCO and KnPCO catalyze the oxidation of MpERF-like_2–15_. (A) Mass spectra of MpERF-like_2–15_ peptide (200 μM) incubated with and without MpPCO for 2 min at 20% O_2_ shows a +32 Da increase in the presence of enzyme, consistent with oxidation. Assays were conducted at 25 °C in the presence of 200 μM AtRAP2_2–15_ using 50 mM Tris/HCl, 0.4 M NaCl, 1 mM TCEP, pH 7.5 as buffer. (B) A 1 h end point assay comparing the activity of AtPCO4, MpPCO, and KnPCO toward the MpERF-like_2–15_ peptide. Assays were conducted at 25 °C using 50 μM peptide, 50 mM bis-tris propane, 50 mM NaCl, and 1 mM TCEP, pH 8.0, as buffer. Letters indicate statistically significant differences (one-way ANOVA followed by Tukey test, *p* < 0.0001; *n* = 3).

A 14-mer peptide representing the Cys-initiating N-terminus of this protein was synthesized (CRMNKRLGKGETGL, hereafter termed MpERF-like_2–15_) and incubated with MpPCO to determine whether it is a substrate for cysteinyl dioxygenation. Following incubation with MpPCO for 1 h under atmospheric conditions at 25 °C, UPLC-MS analysis revealed a +32 Da shift in the peptide mass, consistent with Nt-Cys oxidation to Cys-sulfinic acid (Figure 3A). Under these conditions, MpPCO-catalyzed MpERF-like_2–15_ oxidation reached 89.3% (Figure 3B), demonstrating an ability to oxidize a potentially endogenous substrate in a manner similar to that for substrates from *Arabidopsis* (though, as discussed above, this may just represent an ability of MpPCO to sustain activity over a prolonged incubation period). The presence of arginyl-tRNA transferase (ATE) and E3 N-recognin, PROTEOLYSIS (PRT) 6 homologues in the *M. polymorpha* proteome supports the potential for an O_2_ dependent pathway via the Cys/Arg branch of the N-degron pathway. Our data suggest MpERF-like has the biochemical potential to be regulated via this pathway; in vivo studies will be required to confirm whether it is a physiological N-degron pathway target.

Notably, no putative ERF homologues were identified in the *K. nitens* genome, but Blastp searches of the *K. nitens* genome returned ATE and PRT6 homologues, suggesting the potential existence of a functional N-degron pathway. This is consistent with the evolutionary conservation of elements of this pathway, including in most green algae.^15^ Both recombinant AtPCO4 and KnPCO were also able to catalyze oxidation of MpERF-like_2–15_ to 11.6 and 51.4%, respectively (Figure 3B). Interestingly, KnPCO and AtPCO4 show similar levels of activity toward the peptide substrates originating from *Arabidopsis*, but KnPCO shows a higher level of activity than AtPCO4 toward MpHRE-like_2–15_. Physiologically relevant endogenous substrates of KnPCO remain to be identified.

### MpPCO and KnPCO Have the Potential to Act as O_2_-Sensing Enzymes

Having demonstrated the O_2_-dependence of KnPCO and MpPCO activity, we sought to determine whether their rate of activity was dependent on the availability of O_2_ and therefore whether they have the potential to act as O_2_-sensing enzymes in their respective organisms. For this purpose, kinetic studies of their activity were undertaken with both AtRAP2_2–15_ and MpERF-like_2–15_ substrates.

Prior to kinetic analysis, assays were conducted to establish optimal conditions for MpPCO and KnPCO activity. Oxidation of both AtRAP2_2–15_ and MpERF-like_2–15_ peptides was optimal in the presence of 1 mM tris(2-carboxyethyl)phosphine (TCEP) for both enzymes and optimal buffer pH was found to be pH 8 (Supplementary Figure S4), similar to the AtPCOs.^7^ According to the ICP-MS results, neither MpPCO nor KnPCO is fully saturated with a 1:1 ratio of Fe/protein; therefore, we investigated whether supplementation with additional Fe and ascorbate would increase the rate of enzymatic activity, as seen for two of the AtPCOs.^7^ Fe and ascorbate addition in fact reduced the rate of activity in time-course assays up to 10 min (Supplementary Figure S5); therefore, these components were excluded from kinetic assays. Initial rates of PCO activity are therefore calculated per milligram of total active enzyme present, with the proportion of active enzyme inferred from the fraction of Fe-occupied enzyme.

**Figure 4:**
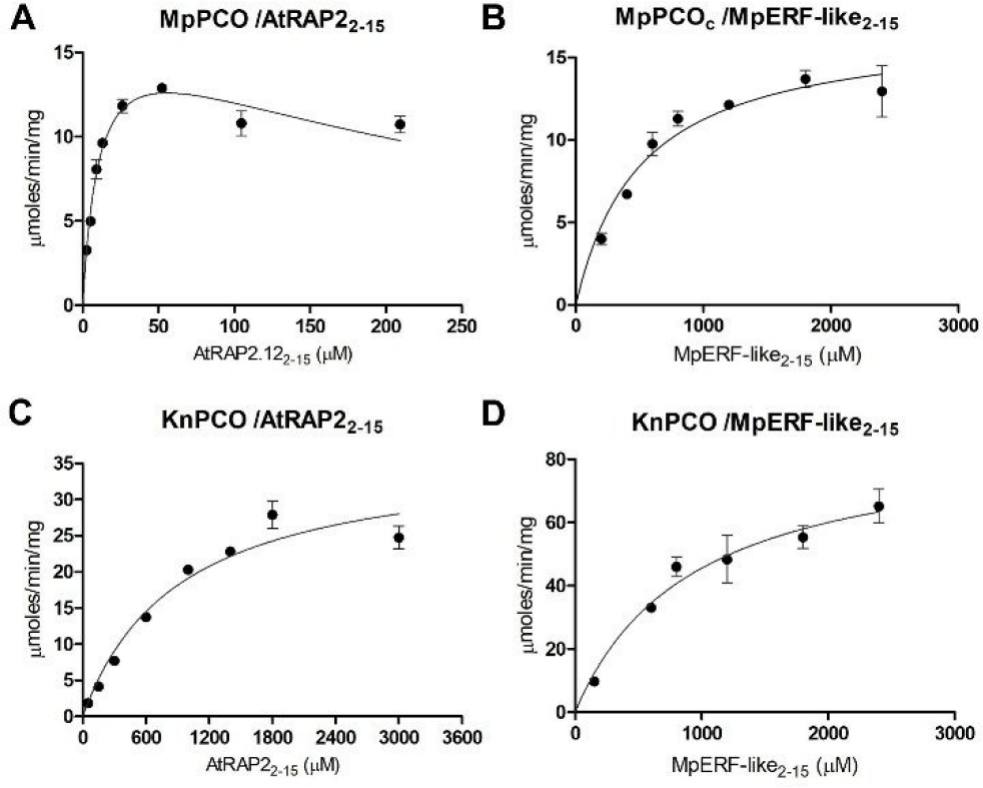
Dependence of MpPCO and KnPCO activity on AtRAP2_2–15_ and MpERF-like_2–15_ availability under atmospheric O_**2**_. Michaelis–Menten kinetic plots for MpPCO and KnPCO activity toward AtRAP2_2–15_ and MpERF-like_2–15_ concentrations are shown. Assays were conducted under aerobic conditions at 25 °C using 50 mM bis-tris propane, 50 mM NaCl, and 1 mM TCEP, pH 8.0, as buffer. Data collected for MpPCO with AtRAP2_2–15_ were fitted to an equation for substrate inhibition to address the decline in rate at higher peptide concentrations. Error bars represent SE (*n* = 3).

We next sought to determine the *K*_M_ of each enzyme for both AtRAP2_2–15_ and MpERF-like_2–15_ substrates in order to ascertain conditions for *K*_M_ (O_2_) assays where peptide substrate concentration was not limiting. In so doing, we noticed that our kinetic data for the reaction of MpPCO with MpERF-like_2–15_ peptide were highly variable in quality when using the high concentrations of peptide necessary for *K*_M_ determination (>1 mM). Light scattering experiments identified that the MpPCO enzyme was more prone to aggregation than a form of the enzyme where the N-terminal His_6_-tag was removed (hereafter MpPCOc, Supplementary Figure S6). MpPCOc did not show variability at high concentrations of MpERF-like_2–15_. Therefore, subsequent kinetic analysis for this reaction used MpPCOc; this form purified with higher levels of Fe (0.36 Fe atoms/molecule, Supplementary Table S1), but as for other enzymes activity was determined for the proportion of Fe-containing enzyme. Initial rates of enzyme activity toward both AtRAP2_2–15_ and MpERF-like_2–15_ substrates were subsequently determined (Supplementary Figure S7) and used to generate Michaelis–Menten plots (Figure 4) and derive kinetic constants (Table 1).

**Table 1:** Steady-State Kinetic Parameters Derived for MpPCO and KnPCO Activity toward AtRAP2_2-15_ and MpERF-like_2-15_^a^

enzyme/substrate	*k*_cat_, s^–1^	*K*_M_, μM	*k*_cat_/*K*_M_, M^–1^ s^–1^	*V*_max_, μmol min^–1^ mg^–1^
MpPCO/AtRAP2_2–15_	8.2 ± 0.5	11 ± 1.1	7.45 x10^5^	17.6 ± 1.1
MpPCO_c_/ MpERF_2–15_	7.9 ± 0.4	509 ± 75	1.55 x10^4^	17.0 ± 0.9
KnPCO/AtRAP2_2–15_	18.0 ± 1.2	914 ± 144	1.97 x10^4^	36.6 ± 2.4
KnPCO/MpERF_2–15_	43.4 ± 4.3	930 ± 223	4.67 x10^4^	87.9 ± 8.7

a Experiments were conducted at atmospheric O_2_.

KnPCO demonstrated a higher rate of activity toward MpERF-like_2–15_, with a turnover (*k*_cat_) of 43.4 s^–1^, compared to 18.0 s^–1^ with AtRAP2_2–15_, which appeared to be linked to greater catalytic efficiency with MpERF-like_2–15_ as *k*_cat_/*K*_M_ values for AtRAP2_2–15_ and MpERF_2–15_ peptides were 1.97 × 10^4^ and 4.67 × 10^4^ M^–1^ s^–1^, respectively. MpPCO was less active than KnPCO in the presence of both substrates, with turnover numbers of 8.2 and 7.9 s^–1^ with AtRAP2_2–15_ and MpERF-like_2–15_, respectively. MpPCO exhibited substrate inhibition in the presence of >50 μM AtRAP2_2–15_; data fitting to a substrate inhibition model indicated an inhibition constant of 0.28 ± 0.06 mM. The *K*_M_ values for MpPCO with the AtRAP2_2–15_ and MpERF-like_2–15_ peptides were 11 and 509 μM respectively; given the similar *k*_cat_ values for these two substrates, this suggests a significantly greater binding affinity of this enzyme for AtRAP2_2–15_ over the (potentially) endogenous MpERF_2–15_. This may be related to the greater proportion of charged residues following the Nt-Cys in MpERF-like_2–15_ (CRMNKRLGKGETGL) compared to AtRAP2_2–15_ (CGGAIISDFIPPPR) impacting the nature of their interaction with MpPCO.

**Figure 5:**
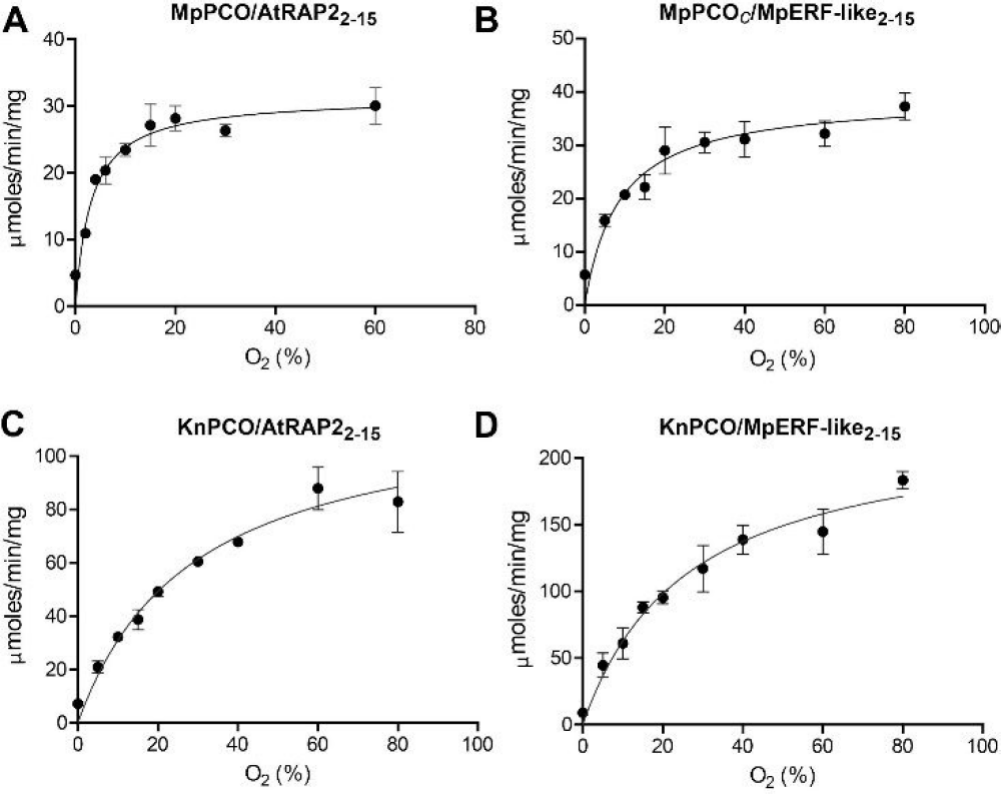
Dependence of MpPCO and KnPCO activity on O_2_ availability. Michaelis–Menten kinetic plots for MpPCO and KnPCO with the AtRAP2.12_2–15_ and MpERF_2–15_ peptides with respect to varying O_2_ availability (solutions saturated with O_2_/N_2_). Assays were conducted at 25 °C using 50 mM bis-tris propane, 50 mM NaCl, and 1 mM TCEP, pH 8.0, as buffer. Peptide concentrations at the point of maximum activity were chosen to ensure turnover was not limited by peptide availability (Figure 4). Error bars represent SE (*n* = 3).

Having identified the optimal concentrations of MpPCO and KnPCO substrate for maximal activity, we next determined their *K*_M_ (O_2_) values to ascertain whether they have the biochemical potential to act as O_2_ sensors. This was done using a method previously described;^22^ briefly, reactions were conducted in sealed vials in which solutions had been saturated with gases at different ratios of O_2_ and N_2_. Reactions were quenched at single time points (either 45 or 60 s) within the known linear rate range (derived from Supplementary Figure S7). Peptide oxidation was quantified by LC-MS analysis, and the data were used to generate Michaelis–Menten kinetic plots from which *K*_M_ (O_2_) values were derived (Figure 5, Table 2). AtPCOs have previously been reported to have *K*_M_ (O_2_) values for AtRAP2_2–15_ ranging from 5.45 to 17.3% O_2_.^8^ Interestingly, MpPCO was found to have a lower *K*_M_ (O_2_) value for this substrate at 3.5% O_2_ and a *K*_M_ (O_2_) value of 8.7% O_2_ for the MpERF-like_2–15_ substrate. The sensitivity of this enzyme to O_2_ availability was therefore rather low compared to the *Arabidopsis* enzymes, suggesting that MpPCO is responsive to changes in O_2_ availability when O_2_ is already below normoxic levels. In contrast, KnPCO had high *K*_M_ (O_2_) values toward both substrates: 28.9 and 26.3% O_2_ with AtRAP2_2–15_ and MpERF-like_2–15_, respectively. These *K*_M_ (O_2_) values are greater than those of any PCO measured to date, suggesting the potential for KnPCO to be highly sensitive to changes in O_2_ availability across a wide range of concentrations.

**Table 2:** Steady-State Kinetic Parameters Derived for MpPCO and KnPCO Activity toward O_2_^a^

enzyme/substrate	*k*_cat_, s^–1^	*K*_M_, %_sat_ (μM)	*V*_max_, μmol min^–1^ mg^–1^
MpPCO/AtRAP2_2–15_	15.5 ± 0.7	3.2 ± 0.6 (39 ± 7)	31.3 ± 1.4
MpPCO_c_/ MpERF_2–15_	18.2 ± 0.9	8.7 ± 1.7 (106 ± 21)	39.1 ± 2.0
KnPCO/AtRAP2_2–15_	59.3 ± 3.7	28.9 ± 4.1 (351 ± 50)	120.2 ± 7.6
KnPCO/MpERF_2–15_	112.2 ± 6.8	26.3 ± 3.8 (319 ± 46)	227.5 ± 13.8

a Experiments were conducted in the presence of nonlimiting AtRAP2_2-15_ and MpERF-like_2-15_.

## Discussion

In flowering plants, the PCOs have been shown to regulate the genetic response to chronic hypoxia such as those observed during developmental processes^13,14^ and acute hypoxia incurred as a result of submergence.^6^ PCO-like sequences are ubiquitous throughout the plant kingdom, however the ERF-VII and other known MC-initiating PCO substrates are confined to the flowering plants, with no homologous ERF-like sequences found in algae. Nevertheless, we and others^15^ have identified putative components of the N-degron pathway, PCO, PRT6, and ATE1 homologues, in the *M. polymorpha* and *K. nitens* proteomes and genomes, respectively. This suggests that early land plants and algae also have the potential to regulate Nt-Cys initiating protein stability in an O_2_-dependent manner. We have investigated the function of putative PCO enzymes from these organisms. We found that they do indeed act as Nt-Cys dioxygenases and that their activity is sensitive to O_2_ availability, particularly that of KnPCO. This means they have the biochemical potential to act as O_2_-sensors. This is the first report of functional PCO enzymes from algae and these findings support an evolutionarily important role for these enzymes.^20^

MpPCO and KnPCO have been characterized with respect to their activity toward a peptide representing the N-terminus of a known PCO target from *Arabidopsis*, the ERF-VII transcription factor RAP2.12 (AtRAP2_2–15_). We also investigated the activity of both enzymes toward two other PCO substrates which have been identified in *Arabidopsis*, ZPR2 and VRN2, and compared their activity to that of AtPCO4. Interestingly, over the course of 1 h of incubation, all three PCO enzymes showed relatively high levels of activity toward AtRAP2_2–15_. However, AtPCO4 and KnPCO showed much lower levels of activity toward AtZPR2_2–15_ and AtVRN2_2–15_. This indicates that, under the conditions used, either oxidation of these substrates by AtPCO4 and KnPCO is much slower than oxidation of RAP2_2–15_ or AtPCO4 and KnPCO are inactivated during the course of this reaction, possibly by substrate or product inhibition. In contrast, oxidation of AtZPR2_2–15_ and AtVRN2_2–15_ was high in the presence of MpPCO. None of these substrates is endogenously present in either *M. polymorpha* or *K. nitens*, meaning these results are not physiologically relevant. However, they do indicate that MpPCO possesses structural features which engender different catalytic and/or substrate binding properties to those of KnPCO and AtPCO4. Although the structural nature of the interaction between PCOs and their substrates is not yet known, structural features of the PCOs^18,21^ and the human thiol dioxygenase ADO^22^ suggest that substrates of these enzymes are likely to bind in an extended manner with little secondary structure, at least in the initial N-terminal region. Substrate sequence recognition by each enzyme (e.g., via a potential substrate-binding flexible loop region near the active site^18^) is therefore likely to contribute to catalytic efficiency. Notably, this β9-β10 loop region in MpPCO lacks negatively charged residues which are present in both AtPCO4 and KnPCO (Supplementary Figure S1). In a physiological context, these differing structural interactions are likely to be important for the endogenous function of MpPCO and could indicate that, despite a low *K*_M_ (O_2_), sustained (albeit slow) activity of this enzyme could nevertheless result in significant levels of substrate oxidation.

As well as demonstrating MpPCO and KnPCO function toward a substrate from *Arabidopsis*, we have identified a putative transcription factor from the *M. polymorpha* proteome which we termed MpERF-like. MpERF-like has a N-terminal Cys residue but does not have the conserved (M)CGGAI motif synonymous with ERF-VIIs in flowering plants.^3^ Nevertheless, we found that MpPCO, KnPCO and also AtPCO4 could all catalyze oxidation of MpERF-like_2–15_. As observed for the *Arabidopsis*-derived substrates, MpPCO sustained activity toward this peptide for 1 h while AtPCO4 and, to a lesser extent, KnPCO showed reduced activity. In steady state kinetic assays, measured under initial rate conditions, KnPCO demonstrated a higher *k*_cat_ value for MpERF-like_2–15_ than it did for AtRAP2_2–15_ while the activity of both enzymes appeared to be O_2_-sensitive with each substrate.

*K*_M_ (O_2_) values can act as indicators of O_2_-sensitivity, as they reflect the relationship between O_2_ availability and rate of enzyme activity; a low *K*_M_ (O_2_) indicates that the rate of enzyme activity will be sensitive to change at low O_2_ concentrations, whereas a high *K*_M_ (O_2_) indicates that the rate of enzyme activity will be sensitive to change across a wider range of O_2_ concentrations. Depending on the physiological O_2_ variations experienced, the ability of an enzyme to “sense” a drop in O_2_ availability (from normoxia to hypoxia) via reduced enzyme activity indicates its potential to act as an O_2_ sensor.

While both KnPCO and MpPCO have the biochemical capacity to act as O_2_ sensors, both the MpPCO/AtRAP2_2–15_ and MpPCO/MpERF-like_2–15_ reactions are sensitive at lower O_2_ concentrations, and across a narrower range, compared to the equivalent KnPCO-catalyzed reactions. The low O_2_-sensitivity of MpPCO may be physiologically relevant, and it is tempting to speculate that this is related to its poorly oxygenated ecological niche. However, it is possible that PCO O_2_-sensitivity is substrate dependent and oxidation of validated endogenous substrates in *M. polymorpha* may prove to have higher *K*_M_ (O_2_) values. Furthermore, MpPCO activity toward 14-mer peptides may not be representative of endogenous activity toward full length proteins. It will be interesting to see which proteins are genuinely regulated by the N-degron pathway in *M. polymorpha* and confirm the O_2_-sensitivity of this process. Conversely, the activity of KnPCO toward both AtRAP2_2–15_ and AtERF-like_2–15_ showed O_2_-sensitivity (as determined by *K*_M_ (O_2_) values) greater than those reported for the AtPCOs.^7^ This intriguing result suggests that, if functional at an endogenous level, the N-degron pathway in *K. nitens* could be highly regulated by O_2_ availability. It also raises the possibility that structural differences between two enzymes, KnPCO and MpPCO, lead to significant differences in O_2_-sensing capability.

Overall, this work confirms that *K. nitens* and *M. polymorpha* have the potential for functional O_2_-sensing enzymes which may regulate protein stability in an O_2_-dependent manner. While the work is conducted at the biochemical level, and thus does not provide direct evidence for endogenous PCO function in these organisms, we nevertheless demonstrate that such function is a possibility. Our results suggest that further investigation into the role of PCO function in early land plants and algae may reveal novel regulatory features and potentially uncover pathways with differing sensitivity to O_2_. Exploring the adaptation and potential levels of O_2_ triggers of stress responses in these simple photosynthetic organisms could reveal how different O_2_ tolerances emerge depending on ecology and/or complexity and could unveil some of the first steps to the evolution of O_2_-sensing function in higher plants.

## Supporting Information Available

Materials and methods; alignment of AtPCO4, MpPCO and KnPCO sequences; gel showing purification of recombinant MpPCO and KnPCO and EPR spectra demonstrating similarity in Fe(III) status with AtPCO4; tandem MS/MS data indicating modification of AtRAP2_2–15_ at N-terminal Cys; determination of optimal assay conditions for MpPCO and KnPCO; determining metal dependence of MpPCO and KnPCO; thermal stability of His6-MpPCO and MpPCOc; initial rate data for MpPCO and KnPCO with AtRAP2_2–15_ and MpERF-like_2–15_, used for kinetic values in Figure 4; metal content of purified proteins (PDF)

## Abbreviations

PCO: plant cysteine oxidase
ERF: ethylene response factor
VRN2: Vernalisation 2
ZPR2: Little Zipper 2
ADO: 2-aminoethanethiol dioxygenase
ICP-MS: inductively coupled plasma mass spectrometry
TCEP: tris(2-carboxyethyl)phosphine
