# Supplementary material for: Plant Cysteine Oxidase Oxygen-Sensing Function Is Conserved in Early Land Plants and Algae
Source: ACS Bio Med Chem Au. 2022 Aug 15;2(5):521–8. doi: 10.1021/acsbiomedchemau.2c00032 (PMC9585510; doi:10.1021/acsbiomedchemau.2c00032)
Supplement: Supplementary file 1 — bg2c00032_si_001.pdf [file bg2c00032_si_001.pdf]

Supplementary Information

Plant Cysteine Oxidase oxygen-sensing function is conserved in early land plants and algae

Leah J. Taylor-Kearney[a,†], Samuel Madden[a], Jack Wilson[a,‡], William K. Myers[b], Dona M. Gunawardana[a], Elisabete Pires[a], Philip Holdship[c], Anthony Tumber[a], Rosalind E. M. Rickaby[c] and Emily Flashman[a*].

[a] Chemistry Research Laboratory, University of Oxford, 12 Mansfield Road, Oxford, OX1 3TA, United Kingdom

[b] Inorganic Chemistry Laboratory, University of Oxford, South Parks Road, Oxford, OX1 3QR, United Kingdom

c Department of Earth Sciences, University of Oxford, South Parks Road, Oxford OX1 3AN, United Kingdom

* Corresponding author: emily.flashman@chem.ox.ac.uk

KEYWORDS enzyme kinetics, evolution, hypoxia, N-degron pathway, oxidase, post-translational modification.

Supplementary Information:

Materials and methods

Supplementary Figures:

**Figure S1**	Sequence similarity for AtPCO4, MpPCO and KnPCO.
**Figure S2**	Purification of recombinant MpPCO and KnPCO
**Figure S3**	Tandem MS/MS demonstrating modification of $RAP2_{2\text{-}15}$ at N-terminal Cys
**Figure S4**	Determining the optimum assay conditions for MpPCO and KnPCO
**Figure S5**	Determining the metal dependence of the MpPCO and KnPCO assays
**Figure S6**	His6-MpPCO is more prone to aggregation than $MpPCO_c$.
**Figure S7**	Initial rate data for KnPCO and MpPCO with different concentrations of $AtRAP2_{2\text{-}15}$ or MpERF-$like_{2\text{-}15}$

Supplementary Tables:

**Table S1**	Metal content of MpPCO, $MpPCO_c$ and KnPCO

Supplementary References

Materials and Methods:

Expression and Purification of MpPCO and KnPCO

The gene encoding MpPCO was amplified from initial constructs donated by Professor Francesco Licausi and cloned into pET28a (Novagen). The gene encoding KnPCO was synthesised and inserted into pET28a (Genscript). Recombinant proteins were expressed and purified as described previously [1]. Briefly, enzymes were expressed in *E. coli* BL21 (DE3) competent cells induced with 0.5 mM isopropyl 1-thio-β-D-galactopyranoside for 16 hours 18 °C. Soluble protein was purified via Ni-affinity chromatography and size exclusion chromatography. Protein purity was assessed by SDS-PAGE.

Peptide Synthesis and Purification

Peptides were either purchased from GL Biochem (China) ($AtRAP2_{2\text{-}15}$ CGGAIISDFIPPPR, $AtZPR2_{2\text{-}15}$ CLTTSEPPFPDTDT, $AtVRN2_{2\text{-}15}$ CRQNCRAKSSPEEV) or synthesised ($MpERF\text{-}like_{2\text{-}15}$, CRMNKRLGKGETGL) using a Liberty Blue™ Microwave Peptide Synthesiser (CEM Corporation), via fluorenylmethyloxycarbonyl (Fmoc) solid phase peptide synthesis using a NovaPEG rink amide resin (Merck). The 14-mer peptides were prepared as C-terminal amides and cleaved from the resin using trifluoracetic acid/water/triisopropylsilane/dimethoxy benzene mix. Peptides were purified using high-performance liquid chromatography and a C18 column.

MpPCO/KnPCO metal content determination

Trace element analyses of metal content in enzyme samples were conducted using inductively coupled plasma mass-spectrometry (ICP-MS) on a NexION 350D ICP-MS (PerkinElmer) coupled with a prepFAST Flow Injection Automation System autosampler (Elemental Scientific). The instrument was calibrated from a series of several standards, which were robotically prepared by the autosampler. Each solution was injected into the instrument nine times at 100ms intervals, and the concentration calculated from the average peak height of the nine injections by comparison to the calibration response curve. Rhodium, indium iridium and rhenium were also added into each measured solution as internal standards, to correct for any instrumental drift that may be caused by matrix suppression. Ratios of Fe per protein molecule were calculated.

KnPCO/MpPCO activity assays

Unless indicated otherwise, the activities of KnPCO and MpPCO were examined by incubating synthesized peptide with 0.1 µM enzyme at 25 °C under aerobic conditions in the presence of 1 mM TCEP, 50 mM Bis tris propane and 50 mM NaCl at pH as specified in the text. Reactions were quenched by the addition of 1% formic acid. For qualitative analysis, oxidation was monitored by ultrahigh-performance LC (UPLC)-MS using an Acquity UPLC system coupled to a Xevo G2-S Q-ToF mass spectrometer (Waters) operated in positive electrospray mode. Instrument parameters, data acquisition and data processing were controlled by Masslynx 4.1 with source conditions adjusted to maximise sensitivity and minimise fragmentation. Samples were injected on to a Chromolith Performance RP-18e 100^{-2} mm column (Merck) heated to 40 ^{o}C

and eluted at 0.3 mL/min using a gradient of 95 % deionized water supplement with 0.1 % (v/v) formic acid to 95 % acetonitrile.

High-throughput steady-state kinetic assays were conducted as described above but were analysed using a RapidFire RF 365 high-throughput sampling robot (Agilent) attached to an iFunnel Agilent 6550 accurate mass quadrupole time-of-flight (Q-TOF) mass spectrometer operating in the positive ionization mode with the parameters: capillary voltage (4000 V), nozzle voltage (1000 V), fragmentor voltage (365 V), gas temperature (225 °C), gas flow (13 L/min), sheath gas temperature (350 °C), sheath gas flow (12 L/min). Samples were aspirated under vacuum for 0.4 s and loaded onto a C4 solid phase extraction (SPE) cartridge at a flow rate of 1.25 mL/min. The C4 SPE was then washed with aqueous 0.1% (v/v) formic acid in LCMS grade water for 5.5 s at a flow rate of 1.25 mL/min followed by elution from the C4 SPE with 85% (v/v) acetonitrile, 15% (v/v) LCMS water containing 0.1% (v/v) formic acid at a flow rate of 1.5 mL/min for 5.5 s. Peptide oxidation was quantified using RapidFire Integrator software (Agilent), where the charged ion with the highest intensity was chosen for deconvolution and peptide quantification. Turnover was quantified by comparing the areas underneath the product and substrate ions. All spectra were assessed manually in Masshunter Qualitative Analysis B.07.00 (Agilent) to ensure the correct ion was chosen for quantification. All figures and kinetic parameters were generated using Prism (GraphPad).

Determining the O_2 Dependence of KnPCO and MpPCO

The activities of KnPCO and MpPCO were examined at different O_2 concentrations using a previously described method [22]. Briefly, silicone-sealed vials containing the desired concentration of peptide and TCEP were equilibrated with different ratios of N_2 and O_2 gas (to give final defined % O_2 concentrations) for 10 min using a mass flow controller (Brooks Instruments). Reactions were initiated by injecting 1.5 µL of enzyme using a gas-tight syringe (Hamilton), incubated at 25 °C for 45-60s and quenched with 80 µL of 1% (v/v) formic acid. The period of incubation was selected based on parameters where it is known that the enzyme is catalysing peptide oxidation under initial (linear) rate conditions (typically from previous kinetic measurements to determine dependence of rate on peptide substrate concentration). Peptide oxidation was analysed by RapidFire Q-TOF MS as described above.

Supplementary Figures

A

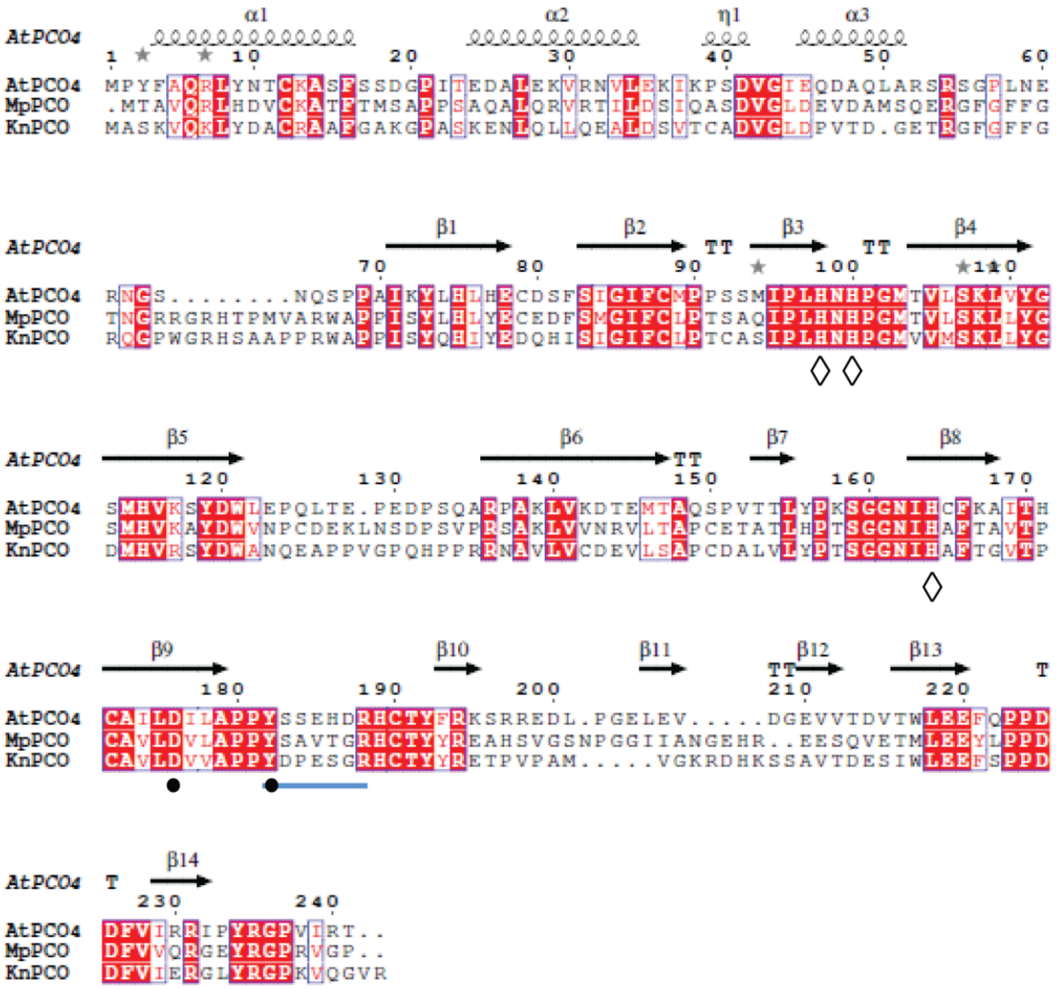

B

Identity Matrix (%)

	AtPCO1	*AtPCO2*	*AtPCO3*	*AtPCO4*	*AtPCO5*	*MpPCO*	*KnPCO*
AtPCO1	100.00	57.61	38.02	46.93	45.41	46.98	43.48
AtPCO2	57.61	100.00	41.57	42.04	40.97	43.36	40.27
AtPCO3	38.02	41.57	100.00	41.35	39.50	43.85	41.32
AtPCO4	46.93	42.04	41.35	100.00	76.76	49.58	42.68
AtPCO5	45.41	40.97	39.50	76.76	100.00	47.28	40.83
MpPCO	46.98	43.36	43.85	49.58	47.28	100.00	54.25
KnPCO	43.48	40.27	41.32	42.68	40.83	54.25	100.00

Figure S1. Sequence similarity for AtPCO4, MpPCO and KnPCO. (**A**) Sequences were aligned using Clustal Omega[2] and conservation annotated using ESPript,[3] with secondary structure assigned based on AtPCO4, PDB ID 6S7E.[4] Residues which are conserved in all sequences have a red background, residues which are similar across

all sequences have red font on a white background. Active site metal-coordinating histidines are indicated with a ◊, conservation of residues important for activity (AtPCO4 Asp176 and Tyr182[4]) are indicated with a ●. Blue line indicates β9-β10 potential substrate binding loop. (**B**) Sequence identity matrix for all AtPCOs compared with MpPCO and KnPCO.

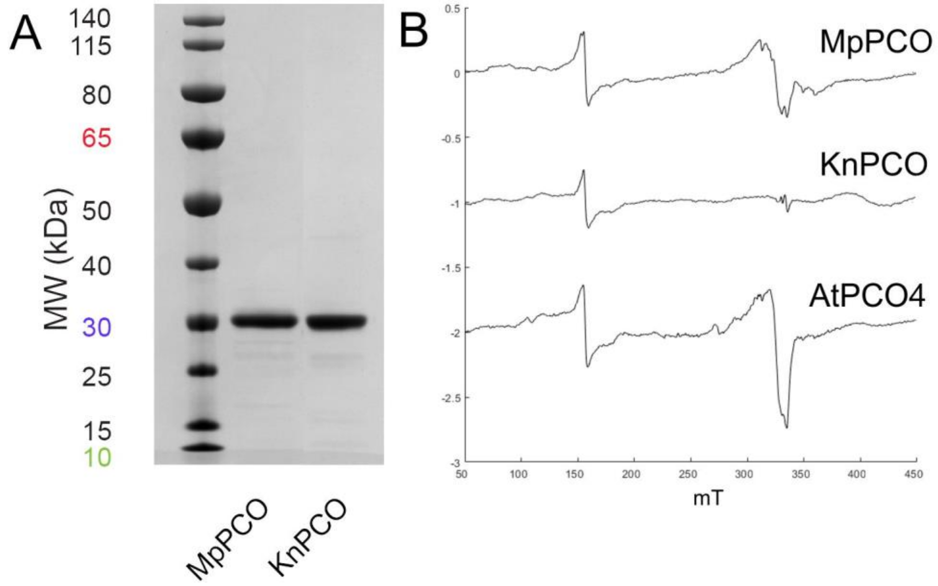

Figure S2. Purification of recombinant MpPCO and KnPCO. (**A**) MpPCO and KnPCO were prepared to >95% purity as judged by SDS-PAGE (expected molecular weights 29753 Da and 29596 Da, respectively). (**B**) Continuous wave EPR spectra of MpPCO, KnPCO and AtPCO4 (all at 100 μM), reveal that all 3 enzymes have similar levels of Fe(III) content (as indicated by signal at 150 mT). Spectra were measured at 5K and a frequency of 9.39 GHz with a modulation amplitude of 4.9 mT and a microwave power of 6.3 mW in a Bruker EMX spectrometer.

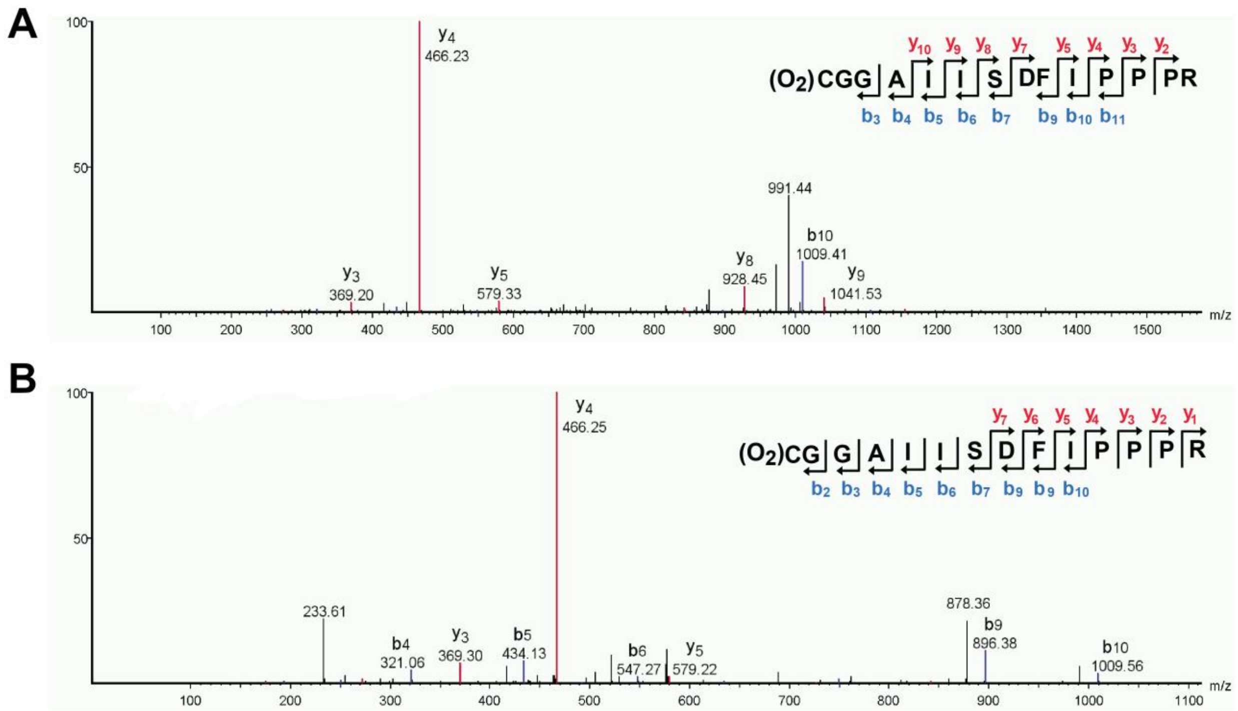

Figure S3: Tandem MS/MS indicating oxidation of AtRAP2$_{2-15}$ at N-terminal Cys. AtRAP2$_{2-15}$ incubated with (**A**) MpPCO or (**B**) KnPCO under 20% O_2 showed a +32 Da modification consistent with oxidation. Tandem MS/MS revealed oxidation likely occurs at the N-terminal Cys of RAP2$_{2-15}$; analysis software listed Cys as modification site with confidence for both MpPCO and KnPCO.

Peptides were analyzed on a NanoAcquity-UPLC system (Waters) connected to an Orbitrap Elite mass spectrometer (Thermo Fisher Scientific) possessing an EASY-Spray nano-electrospray ion source (Thermo Fisher Scientific). The peptides were trapped on an in-house packed guard column (75 µm i.d. x 20 mm, Acclaim PepMap C18, 3µm, 100 Å) using solvent A (0.1% Formic Acid in water) at a pressure of 140 bar. The peptides were passed over an EASY-spray Acclaim PepMap® analytical column (75 µm i.d. × 50 mm, RSLC C18, 3 µm, 100 Å) using a linear gradient (length: 100 minutes, 3 % to 60 % solvent B (0.1% formic acid in acetonitrile), flow rate: 200 nL/min). Peptides were electrosprayed directly into the mass spectrometer using a CID based method; analysis was performed with Peaks 8.5. All spectra were manually validated.

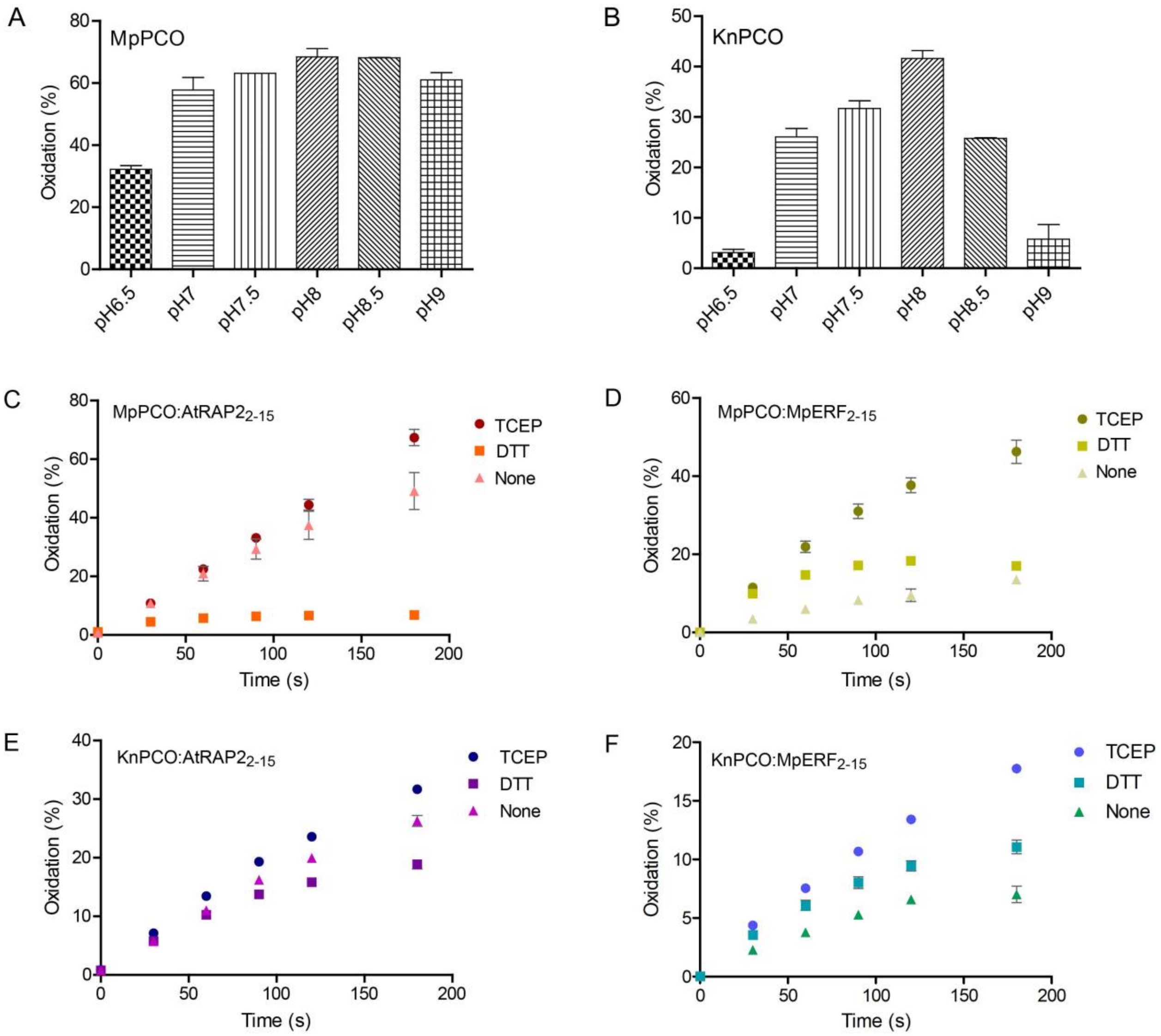

Figure S4: Determining optimum assay conditions for MpPCO and KnPCO. The activity of 0.2 μM (**A**) MpPCO and (**B**) KnPCO towards 100 μM MpERF-like$_{2-15}$ under aerobic conditions after 10 minutes at 25 °C in the presence of 1mM TCEP and 50 mM NaCl/Bis tris propane, at pH values ranging from 6.5 to 9.0 (n=2). Activity of 0.2 μM MpPCO (**C**/**D**) and KnPCO (**E**/**F**) towards 100 μM AtRAP2.12$_{2-15}$ and MpERF-like$_{2-15}$ in the absence or presence of 1 mM DTT or TCEP. Assays were conducted under aerobic conditions at 25°C in a buffer of 50 mM NaCl/Bis tris propane, pH 8.0; n=3. Error bars represent S.E.

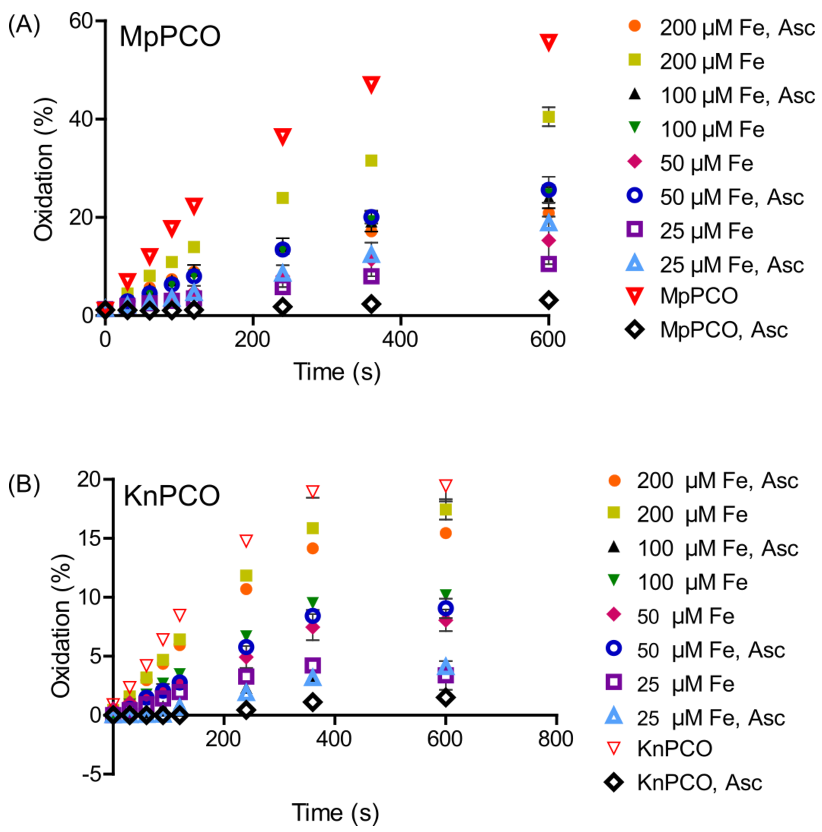

Figure S5: Determining the iron and ascorbate dependence of the MpPCO and KnPCO assays. Activity of **(A)** MpPCO and **(B)** KnPCO towards 100 µM MpERF-like$_{2\text{-}15}$ in the presence of Fe(II) and sodium ascorbate. Assays were conducted under aerobic conditions at 25°C in a buffer of 50 mM NaCl/Bis tris propane, pH 8.0 and 1 mM TCEP; n=3. Fe(II) and ascorbate concentrations were varied. Reactions were quenched in 1% (v/v) formic acid. Error bars represent S.E.

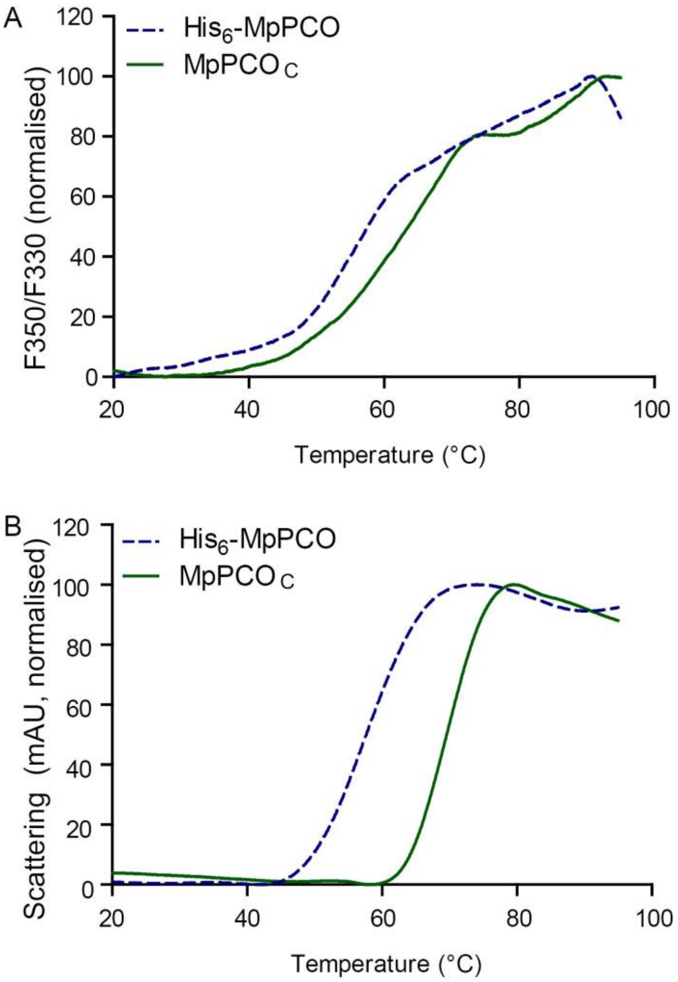

Figure S6: His$_6$-MpPCO is more prone to aggregation than MpPCO$_c$. Representative plots showing (**A**) thermal stability of His$_6$-MpPCO and MpPCO$_c$ proteins which was determined to be similar using the fluorescence ratio 350/330 nm, and (**B**) aggregation temperatures determined using light scattering data. The average onset for scattering was 60.5 ± 2.8 °C and 44.7 ± 2.8 °C for MpPCO$_c$ and His$_6$-MpPCO respectively (n=6), suggesting His$_6$-MpPCO is more prone to aggregation than MpPCO$_c$. Analyses were conducted using a Nanotemper Prometheus NT.48 with proteins at 10 μM; plots represent an average of 6 samples.

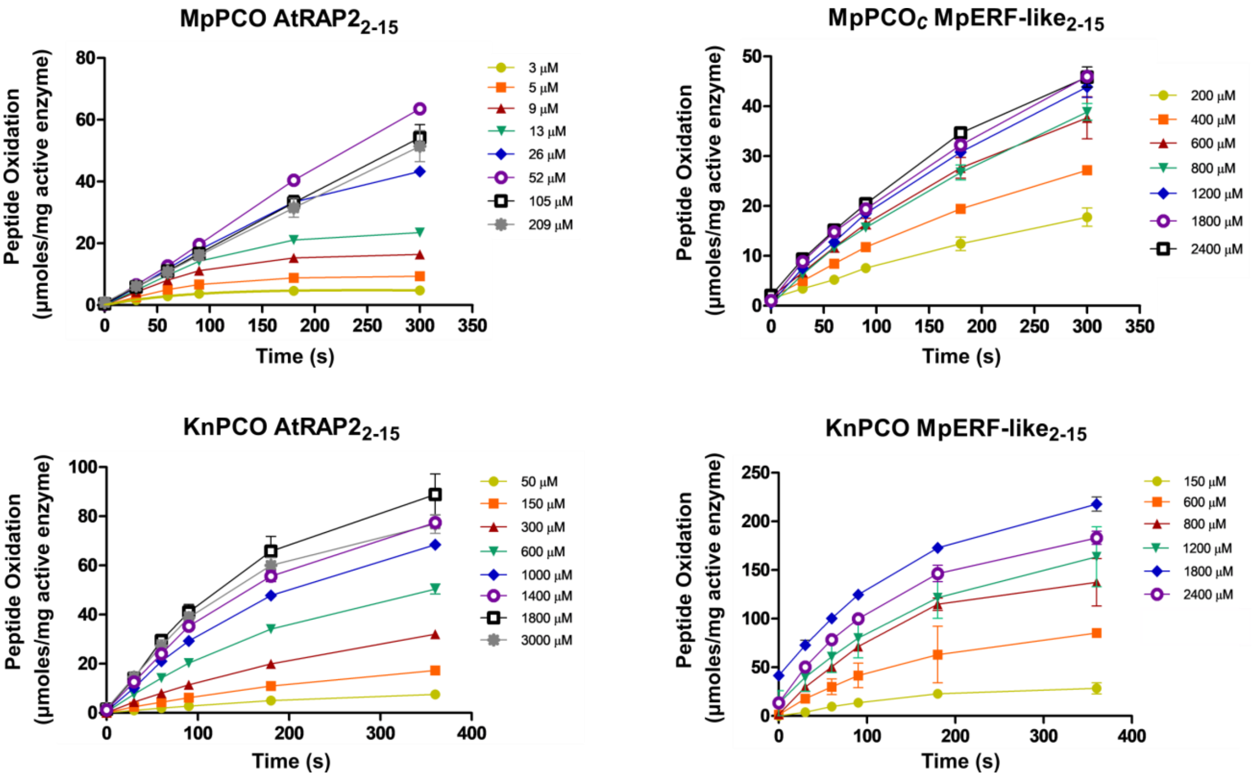

Figure S7: Initial rate data for KnPCO and MpPCO with different concentrations of AtRAP2$_{2\text{-}15}$ or MpERF-like$_{2\text{-}15}$. Rate profiles for MpPCO/MpPCO$_c$/KnPCO with AtRAP2$_{2\text{-}15}$ or MpERF$_{2\text{-}15}$, as indicated, at atmospheric O_2. Assays were conducted at 25 °C using 0.1 µM enzyme (adjusted for Fe(II)-containing proportion) in a buffer of 50 mM Bis tris propane, 50 mM NaCl and 1 mM TCEP, pH 8.0, and analysed by LC-MS. Error bars represent S.E. (n = 3). Connecting lines are presented to illustrate changes in gradient and are not fitted curves. Initial rates were calculated from gradients over the first 0-90 s.

Protein	Bound Metal		
	Fe	Zn	Ni
MpPCO	0.16 (± 0.01)	0.08 (±0.01)	0.4 (± 0.03)
$MpPCO_c$	0.36 (± 0.00)	0.13 (± 0.00)	0.13 (± 0.00)
KnPCO	0.27 (± 0.01)	0.08 (± 0.02)	0.23 (± 0.01)

Table S1: Metal content of MpPCO, $MpPCO_c$ and KnPCO. The metal content of each protein was quantified using inductively-coupled plasma mass spectrometry. All proteins had substoichiometric iron levels, as observed previously for AtPCOs,[1] proportions of which were used to calculate amount of each enzyme that was active. Contaminating Zn and Ni were previously observed for AtPCOs.[5]

Supplementary References

(1) White, M. D.; Kamps, J.; East, S.; Taylor Kearney, L. J.; Flashman, E. The plant cysteine oxidases from Arabidopsis thaliana are kinetically tailored to act as oxygen sensors. *J Biol Chem* **2018**, *293* (30), 11786-11795. DOI: 10.1074/jbc.RA118.003496

(2) Sievers, F.; Wilm, A.; Dineen, D.; Gibson, T. J.; Karplus, K.; Li, W.; Lopez, R.; McWilliam, H.; Remmert, M.; Soding, J.; et al. Fast, scalable generation of high-quality protein multiple sequence alignments using Clustal Omega. *Mol Syst Biol* **2011**, *7*, 539. DOI: 10.1038/msb.2011.75

(3) Robert, X.; Gouet, P. Deciphering key features in protein structures with the new ENDscript server. *Nucleic Acids Res* **2014**, *42* (Web Server issue), W320-324. DOI: 10.1093/nar/gku316

(4) White, M. D.; Dalle Carbonare, L.; Lavilla Puerta, M.; Iacopino, S.; Edwards, M.; Dunne, K.; Pires, E.; Levy, C.; McDonough, M. A.; Licausi, F.; et al. Structures of Arabidopsis thaliana oxygen-sensing plant cysteine oxidases 4 and 5 enable targeted manipulation of their activity. *Proc Natl Acad Sci U S A* **2020**, *117* (37), 23140-23147. DOI: 10.1073/pnas.2000206117

(5) White, M. D.; Klecker, M.; Hopkinson, R. J.; Weits, D. A.; Mueller, C.; Naumann, C.; O'Neill, R.; Wickens, J.; Yang, J.; Brooks-Bartlett, J. C.; et al. Plant cysteine oxidases are dioxygenases that directly enable arginyl transferase-catalysed arginylation of N-end rule targets. *Nat Commun* **2017**, *8*, 14690. DOI: 10.1038/ncomms14690
